# Cutaneous Ulcer as Leading Symptom of Systemic Cytomegalovirus Infection

**DOI:** 10.1155/2015/723962

**Published:** 2015-02-16

**Authors:** Richard F. Guo, Frew H. Gebreab, Emily Hsiang-Ho Tang, Zhe Piao, Steve S. Lee, Mario L. Perez

**Affiliations:** ^1^Department of Internal Medicine, Southern California Permanente Medical Group, Fontana, CA 92335, USA; ^2^Department of Dermatology, Southern California Permanente Medical Group, Fontana, CA 92335, USA; ^3^Department of Pathology, Southern California Permanente Medical Group, Fontana, CA 92335, USA; ^4^Department of Rheumatology, Southern California Permanente Medical Group, Fontana, CA 92335, USA; ^5^Department of Infectious Diseases, Southern California Permanente Medical Group, Fontana, CA 92335, USA

## Abstract

Cytomegalovirus (CMV) infection rarely manifests with skin ulcerations. We describe a case report of a 64-year-old woman with chronic immunosuppression for treatment of mixed connective tissue disease, presenting with new onset leg ulcerations after a recent change in immunosuppressive regimen. She subsequently developed fulminant hepatitis, encephalopathy, and pancytopenia and was found to have severe systemic CMV viremia. Skin ulcer biopsy was positive by immunohistochemical staining for CMV infected endothelial cells. Both systemic disease and skin ulcer rapidly improved after stopping immunosuppression and administering intravenous ganciclovir. New onset skin ulcers in an immunosuppressed individual, especially with recent changes in immunosuppressive regimen, should raise the suspicion of reactivation of CMV.

## 1. Introduction

Cytomegalovirus (CMV) is a latent infection in most exposed individuals [1]. In the context of immunosuppression, the virus can reactivate and produce profound systemic disease [2]. CMV has been implicated in encephalitis, retinitis, esophagitis, mucositis, hepatitis, and bone marrow suppression [3–5]. The presentation can be diffuse and nonspecific, and diagnosis is often delayed as a result. Serological testing is only partially useful due to delays in seroconversion and the difficulty in differentiating between latent disease and reactivation. The advent of PCR has improved diagnostic accuracy, offering a highly sensitive and specific serum-based method of detecting and quantifying active disease [6]. Current recommendations regarding therapy are well-established, with favorable outcomes seen with intravenous (IV) ganciclovir.

## 2. Case

A 63-year-old woman with a history of mixed connective tissue disease (MCTD), interstitial lung disease, severe pulmonary hypertension, and paroxysmal atrial fibrillation was admitted for a one-day history of jaundice and a two-week history of malaise and confusion. The patient's MCTD had historically been controlled with azathioprine and prednisone. Six weeks prior to presentation, the patient's rheumatologist increased the dosage of prednisone from 2 mg daily to 40 mg daily due to worsening myositis. One month after the increase in steroid dose, the patient noticed worsening pedal edema and the development of large bullae in the bilateral lower extremities that subsequently ruptured and left behind ulcers. Two weeks later, the patient's family noticed increasing confusion along with somnolence and brought the patient to the emergency department for evaluation.

**Figure 1:**
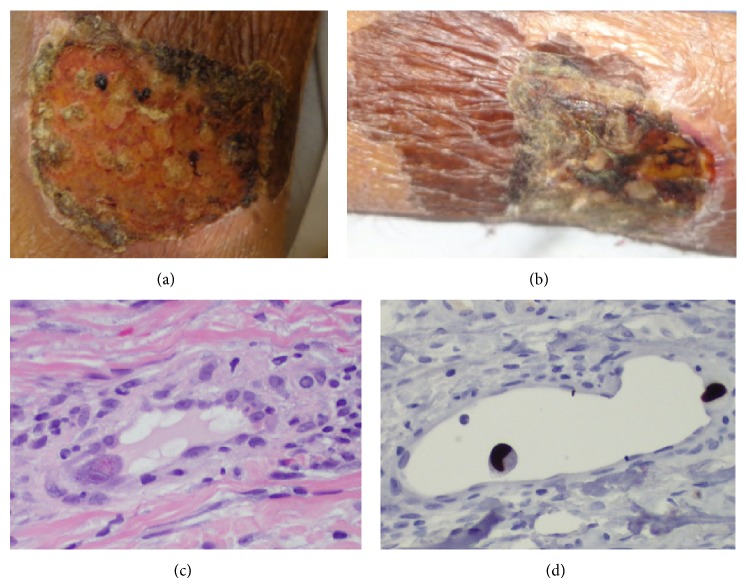
(a) Lower leg ulcer on admission, (b) lower leg ulcer after five days of ganciclovir treatment, (c) H&E staining showing cytoplasmic and nuclear inclusions characteristic of CMV infection, and (d) immunohistochemistry staining for CMV.

On admission, temperature was 36.7°C (98.0 F), blood pressure 124/98 mmHg, and regular pulse of 69 beats per minute. Respiratory rate was 22 breaths per minute, and oxygen saturation was 97% on room air. Pertinent physical exam findings include marked scleral icterus, jaundice, diffuse abdominal tenderness, a nonverbal state, and two symmetric well-circumscribed oval shallow ulcerations on bilateral lower medial legs (Figure 1(a)). Laboratory findings were significant for a white blood cell (WBC) count of 900 per cubic millimeter (82% N, 13.8% L, 1.0% M, 1.3% E, and 1.9% B, ANC 700 per cubic mm), hemoglobin of 12.8 g/dL, and platelet count of 155,000 per cubic millimeter. Aspartate transaminase (AST) and alanine aminotransferase (ALT) were 83 and 144 units per liter, respectively. Total serum bilirubin count was elevated at 24.2 mg/dL. International normalization ratio (INR) was 8.8 on chronic warfarin therapy, which the patient was taking due to atrial fibrillation. Serum ammonia was 42 *μ*g/dL. Urinalysis showed mild proteinuria and 11–25 leukocytes per high power field. Blood culture was positive for aerobic gram negative rods on two sequential tubes. Abdominal ultrasound showed cholelithiasis without biliary dilation. Computed tomography of the abdomen showed a small enhancing lesion in the medial segment of the left hepatic lobe.

Initially, the patient was diagnosed with septicemia, cholestatic jaundice, hepatic encephalopathy, and neutropenia. She was treated with ceftazidime, ciprofloxacin, and lactulose. Warfarin was not resumed. Azathioprine was stopped due to concern of causing bone marrow suppression and contributing to hepatic toxicity. Prednisone dose was tapered off. Other causes of liver injury were investigated, with hepatitis A IgM, hepatitis B surface antigen, hepatitis B and C polymerase chain reaction (PCR), anti-mitochondrial antibody, and anti-smooth muscle antibody all returning negative.

In the subsequent hospital days, the patient's liver markers remained stably elevated. Her mental status gradually worsened, with unresponsive episodes at times. She later developed worsening right heart failure with systemic congestion. Brain magnetic resonance imaging and electroencephalography were performed, which did not explain the etiology of encephalopathy. The gram negative rod was eventually speciated as extended-spectrum-beta-lactamase (ESBL)* Escherichia coli*, so the antibiotic regimen was switched to meropenem.

On hospital day six, WBC count was 600 per cubic millimeter and platelet count was 90,000 per cubic millimeter. Total serum bilirubin was 25.7 mg/dL. Cytomegalovirus (CMV) DNA PCR of serum returned with 173,953 copies. CMV serum IgM avidity index (AI) was elevated at 2 (lab threshold of 1.1 AI for positive antibody) and IgG level was elevated at an AI of 36.3 (lab threshold of 1.1 AI for positive antibody). There were no prior levels for comparison. The patient was started on IV ganciclovir at 5 mg/kg every 12 hours. Subsequently, the patient had rapid and marked improvement in laboratory markers and mentation. Two days after initiating ganciclovir, she was able to speak a few words and follow basic commands. Leg ulcerations improved rapidly and significantly (Figure 1(b)). Total serum bilirubin count decreased to 17.2 mg/dL after four days of treatment. WBC count recovered to 2,000 per cubic millimeter and platelets to 221,000 per cubic millimeter after six days of therapy.

Shave biopsy of the left lower leg legion was performed on hospital day five, prior to initiation of IV ganciclovir. The formalin-fixed, paraffin-embedded tissue samples were cut into 3 to 4 *μ*m thick sections and the slides were then subjected to heat-induced epitope retrieval methods. Immunohistochemistry was performed with an automated immunohistochemistry staining system (Ventana BenchMark ULTRA). A polymer-based method using the ULTRAVIEW Universal DAB detection kit (Ventana) with a commercially purchased primary antibody against cytomegalovirus (Clones DDG9 and CCH2) was used. Appropriate positive and negative controls were used. Using this protocol, histopathology showed scattered endothelial cells with eosinophilic cytoplasmic inclusions and a characteristic halo surrounding the nuclear inclusion (Figure 1(c)). Immunohistochemistry was positive for CMV inclusions (Figure 1(d)). Staining was negative for bacterial and fungal elements. No perivascular infiltration of leukocytes was seen. No other causative etiology of ulceration was seen on pathology. Subsequent ophthalmologic evaluation was negative for retinal involvement due to CMV.

## 3. Discussion

CMV is a member of the herpes family of viruses that infects initially through exposure to bodily fluids containing virions, with the most common routes being through saliva, breast milk, urogenital secretions, and blood [7, 8]. After initial infection, the virus remains latent within monocytes [1] until reactivation is triggered by immunosuppression due to HIV, medications, or stress [2, 9]. It is estimated that the population prevalence of CMV latency ranges from 47 to 81% [10]. Reactivation, usually in the setting of immunosuppression, can cause systemic disease, manifesting as hepatitis [3], retinitis, colitis, pneumonitis, esophagitis [4], and bone marrow suppression [5].

In our case presentation, the patient's CMV serological studies, with elevated IgG levels, likely reflected reactivation of latent disease rather than newly acquired infection. Reactivation has been shown to be strongly correlated with TNF-*α* level [1]. The presence of wound healing with increased local inflammation and elevated TNF-*α* levels, combined with recent increase in immunosuppression, presented the ideal situation for CMV reactivation. This is further assisted by local macrophage infiltration into the granulation tissue, which serves as host for viral reactivation. Subsequently, virions disseminate hematogenously to cause systemic disease. We suspect in our patient that there was a systemic increase in TNF-*α* levels due to concurrent gram negative septicemia, which can assist in the widespread dissemination of CMV-positive mononuclear cells [11, 12]. We believe that the skin lesion reflects the initial focus of CMV reactivation due to the clinical timeline. In the literature, cutaneous CMV lesions have been noted to precede systemic infection [13], and in our patient this was the first symptom after an increase in immunosuppression. As further temporal correlation, the patient had rapid healing of the ulcer after initiation of IV ganciclovir, and histopathology of the ulcer did not reveal any other causative etiology.

Cutaneous ulcers, when present, should evoke a broad differential of infectious and noninfectious agents. The most common causes, especially in the elderly patient, are chronic venous stasis, pressure, and arterial insufficiency. Also important to consider is malignancy, such as basal cell carcinoma, squamous cell carcinoma, and cutaneous lymphoma. Infectious causes include histoplasmosis, blastomycosis, leishmaniasis, staphylococcus, and streptococcus, among others. Due to this broad differential, a biopsy of the ulcer is often required for definitive diagnosis. In the immunocompromised patient in particular, special care should be taken to evaluate for human immunodeficiency virus (HIV), herpes simplex virus (HSV), Kaposi's sarcoma, secondary cutaneous manifestation of fungal infection, and tuberculosis.

Cutaneous ulcers due to CMV are rarely reported in the literature, and the few published reports have been associated with a state of immunosuppression. The exact morphology of skin lesions associated with CMV is variable, including the literature reports of petechiae, nodules, ulcers, and erosions. There does not seem to be a predilection for a particular location, with reports of ulcers occurring in the perineum and heel [14, 15] as well as scrotum and axilla [16].

## 4. Conclusion

There is no established guideline for treatment of cutaneous ulcers due to CMV. Treatment strategies have been extrapolated from existing successful treatment of solid organ CMV infections, with IV ganciclovir and oral valganciclovir [12, 14].

Due to the delay in recognition of systemic CMV infection with a possible focus for reactivation in the skin lesion, our patient's treatment was also delayed. Because CMV has a high prevalence of latency, it is important to consider new cutaneous ulcers in an immunosuppressed patient as a possible presentation of severe systemic CMV disease.
